# Temporality of experiencing and overcoming stigma for children born in captivity (CBIC) among the Lango people of Northern Uganda

**DOI:** 10.1007/s44282-025-00329-8

**Published:** 2026-01-27

**Authors:** Eunice Otuko Apio, Sabine Lee

**Affiliations:** University of Birmingham, Birmingham, B15 2TT UK

**Keywords:** Children, Conflict, Sexual violence, Integration, Time, Temporality, Northern Uganda

## Abstract

This article explores the significance of understanding the experiences of time in the reception and integration of children born in captivity (CBIC) into their kinship groups and local communities in Lango in Northern Uganda. The paper adds to research on the integration experiences of children born in captivity (CBIC) during the Lord’s Resistance Army (LRA) and cattle raiding conflicts in Northern Uganda. It considers aspects of temporality in the reception and integration experiences of these children upon returning to their maternal kinship groups after escaping hostilities. Methodologically, the paper is based on a secondary analysis of qualitative data (focus group discussions [FGDs] and interviews), collected as part of a study of ‘Film-in-Participatory Action Learning (FPAL)’, in which the authors explore the effectiveness of film in a development intervention aimed at groups on the margins in northern Ugandan communities, particularly CBIC. FPAL studies critical themes that intersect with the concept of time, and which are shown to impact on the lived realities of children whose births are associated with conflict-related sexual violence (CRSV). The paper argues that themes of space and time emerging from FGDs are illustrative for how CBIC typically experience life and in particular how experiences of stigma and discrimination are rooted in individual and community perspectives of a generational identity that defines an individual as being located in a specific geographical and generational (thus temporal) space. Using the experiences of CBIC and perspectives of members of the local communities within which they are raised, the paper investigates how perceptions and experiences of time affect the individual’s and community’s responses to being a CBIC or living with CBIC. Specifically, it unpacks how ‘generational time’ that links an individual to their ancestry and descendants affects the CBIC’s place within the local kinship group, how CBIC themselves experience the passage of time since they have joined their maternal clans, and how this passage and the different life stages from childhood through adolescence to adulthood with their specific rights and responsibilities within their local communities are understood by the CBIC themselves.

## Introduction

Since the beginning of this century, children born of war (understood as children conceived by foreign soldiers in war, occupation or peacekeeping missions or children of child soldiers have been the focus of increasing scholarly [9, 10, 20, 25, 31, 34] and advocacy [11, 14–18] attention. This includes children conceived in consensual relationships as well as those conceived in more or less exploitative, abusive or even violent relationship [20, 27, 28]. A recent evidence review found that the region most extensively researched with regard to children conceived in conflict-related sexual violence, is Northern Uganda [37], and indeed some of the ground-breaking work on Children Born of Conflict Related Sexual Violence (CBoCRSV) is the work done by Ugandan and international researchers on children born in LRA captivity [2, 7, 12, 22]. The starting point has often been the trauma of the mothers, and the integration challenges of the mothers and their children once they escaped or were released or rescued from captivity. However, while it has been demonstrated, that these experiences were dominated by discrimination, ostracization and stigma, some of the culturally and temporally impacted aspects of this stigma remain poorly understood. It is this temporality as part of an individual’s place in the intergenerational social contract in Uganda, that is the focus of this paper. Our study take as a point of departure children born in captivity (CBIC) in two distinct conflicts that targeted the Lango of Northern Uganda: (i) those born as a result of CRSV committed on thousands of women and girls abducted by the Lord’s Resistance Army (LRA) from among the Lango language group in northern Uganda between 1987 and 2008 and taken to LRA bases, mostly in southern Sudan, where they served as forced wives, porters and fighters [21]; and (ii) children born to women abducted as part of the cattle-raiding conflicts in the border region of Lango and Karamoja.

Extensive literature provides accounts of the formation, conduct of the LRA, and the experiences of those who were voluntarily or forcibly enlisted [1, 24]. In particular, forced marriage, sexual exploitation, abuse and violence in the LRA are well understood, and research has demonstrated, among others, the ideological underpinning of the LRA’s systematic use of forced marriage and’ sexual exploitation as part of their ‘nation building’ efforts [2, 6, 26] which resulted in the births of thousands of CBIC. Mothers who demobilised often returned to their pre-war communities with their CBIC. The norm in the Ugandan cases of the LRA was for the return of the children to be linked directly to the demobilisation of their mothers who returned – often through reception centres - with their CBIC; only in rare instances did CBIC return on their own [3]; a return with their fathers is not described in the literature or evidenced in the datasets of the reception centres. Existing literature instead suggests that in the few occasions the genitors sought a relationship with CBIC, they did so upon reunification post conflict [14, 28]. Our dataset, consistent with this earlier research, did not include any CBIC who reported having returned with, having reunited with or living with their biological father.

The second group is a much less researched group of children born of conflict-related sexual violence perpetuated during the decades-long and ongoing cattle raiding conflict inflicted on the Lango in Northern Uganda by groups of armed raiders from Karamoja in north eastern Uganda [3, 5, 32]. These cattle raids on Lango community and neighbouring communities in Acholi and Teso by heavily militarised groups of warriors were reportedly aided by the National Resistance Army (NRA) that equipped them with light and heavy firearms to target the ‘difficult North that still had pockets of military resistance’.^1^ This particularly protracted conflict of cattle raids, which peeked in the second half of the 1980s, has not received much scholarly attention, but emerging literature associated with the cattle raiding conflict indicates persistent high prevalence of CRSV [3]. Not only did the raiders forcefully take millions of heads of cattle, they also killed civilians and abducted, raped and trafficked children and young women into Karamoja. Many of the girls and women would later serve as forced wives to their captors. Like the forced wives in the LRA, girls and women who escaped their cattle raiding captors often returned to their marital and/or birth communities with children [4].


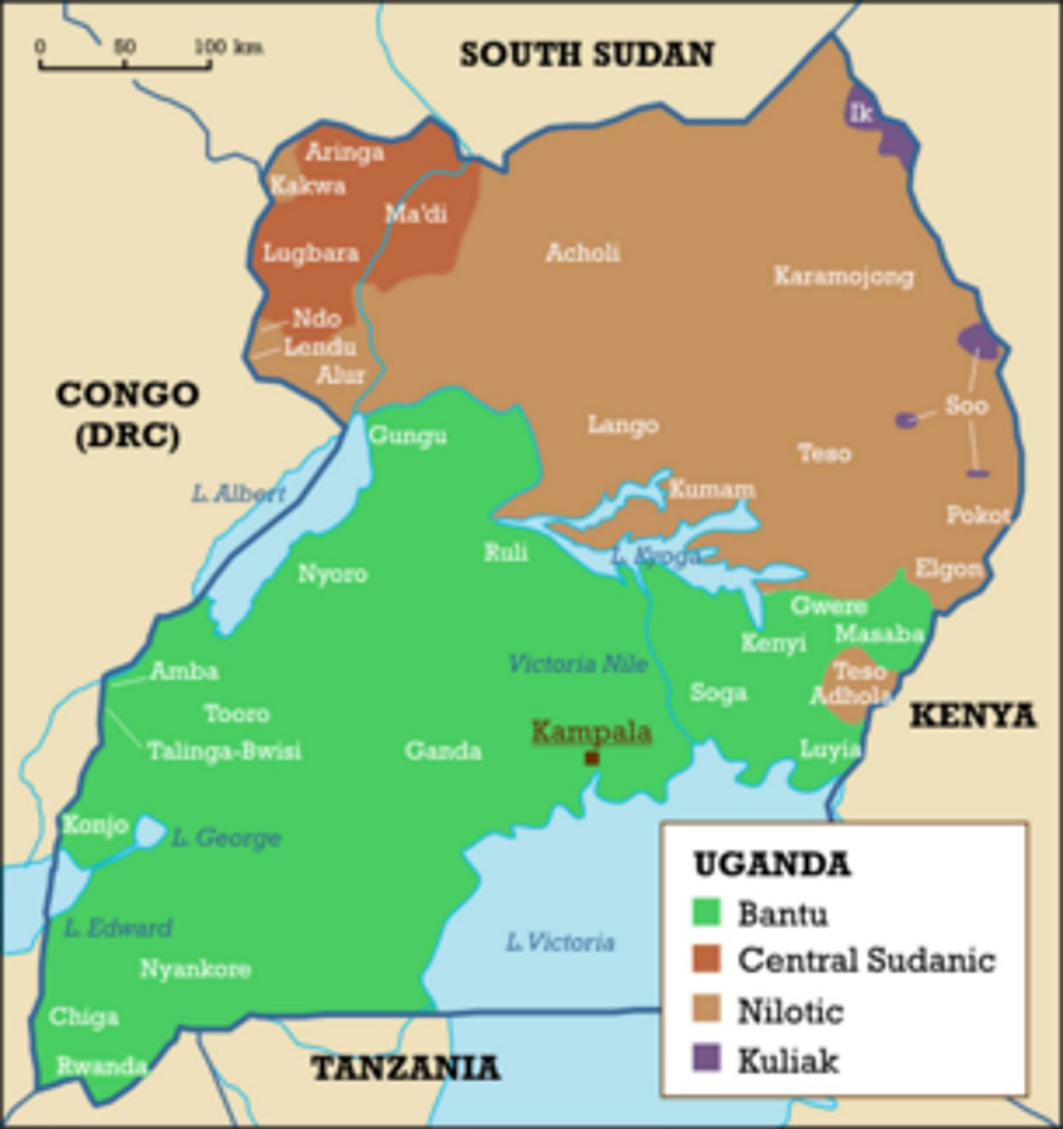


https://en.wikipedia.org/wiki/Language_family.

The significance of time and place, and in particular patterns of generational governance and the cultural and economic significance of generational and kinship contracts with their rights and responsibilities specifically with regard to land and inheritance matters are well understood [2, 13]. Our focus, in contrast, is the generational time in terms of a person’s temporal situatedness within the generational kinship system. Here, we apply Browne’s [8] understanding of generational time as *relational time* to explore how time is produced and how it is lived by CBIC who lived among communities in northern Uganda. We will explore this ‘relational time’ as moments that codify and articulate sense of belonging, enabling sociocultural and political transmission between individuals of different ages and insights, that continually shape experiences of individuals as they make claims and counterclaims, punctuated by rites of kinship, marriage, births and death.

The paper argues that themes of space/time are illustrative of how CBIC typically experience life, and in particular how experiences of stigma and discrimination are rooted in individual and community perspectives of a generational identity that defines an individual as being located in a specific geographical and generational (thus temporal) space. Firstly, the paper briefly presents the methodology. Secondly, it discusses the prevailing Lango kinship system into which the CBIC integrated after they accompanied their mothers from captivity. The paper then explores how not knowing a biological father or their lineage distorted the sense of generational time for CBIC and exposed them to a lack of avenues for claims and counter claims relating to resources that are directly linked to positionality within the generational kinship system. It further investigates how individuals who had already been conceived before their mothers were abducted, and were therefore born either in captivity or upon the return of their mothers after a short period in captivity, had similar experiences of generational time distortion, and were summarily referred to as CBIC. Lastly, it presents the different ways both the CBIC and those they interacted with were often ‘fighting back’ to reorganise and integrate within the prevailing generational time.

## Methodology

The article is based on secondary analysis of a section of data from fieldwork that was collected as part of the Rights for Time^2^-funded ‘Film in Participatory Action Learning (FPAL) study for the integration of children born of war’ from January 2023 to April 2024. This project aimed to increase public knowledge and understanding of the challenges associated with being a CBIC in post-conflict societies, particularly in northern Uganda where this topic remained a taboo and CBIC continue to sit on the margins of society. The F-PAL project design embedded within its qualitative methodology the screening of the film ‘The Wound Is Where the Light enters’ (https://vimeo.com/606439654) as a mobilisation and sensitization tool to inform and change perceptions and practices within communities to improve the wellbeing of CBIC and their families.

For this current article, the segment of data used was drawn from KIIs and FGDs, transcribed and translated to English, and analysed inductively to establish themes and determine how different participants responded around each of the themes. The material was subsequently re-analysed with particular focus on generational time and CBIC experiences.

Firstly, we reviewed a section of data gathered from four (4) Focused Group Discussions (FGDs) conducted with CBIC in Okwang and Adwari sub counties of Otuke district. The FGDs were segregated by sex and all participants were18 years and older. In Okwang, the all-male group comprised 5 participants (two of them with births linked to the LRA and three linked to cattle raiders), while the all-female group had 6 participants (four of them linked to the LRA and two to cattle raiders). In Adwari sub county, the all-male group comprised 5 CBIC linked to cattle raiders, and the all-female group had 6 participants, two of them with births linked to the LRA and the remaining four linked to cattle raiders.

To further gain insights into the local cultural and political underpinnings related to CBIC the study team reviewed data gathered during two FGDs with community members, one in Okwang and the other in Adwari. Participants in Okwang comprised two former forced ‘wives’ whose children had been part of the CBIC Focus Group Discussions, Local Council chairpersons of two villages which had a number of resident CBIC, a local woman leader, a step father to a CBIC, a catechist, and an elder. Participants in Adwari comprised of a stepfather to a CBIC, a clan leader, the deputy head teacher of Adyera Konya Primary School, two village local council chairpersons, the Speaker of Adwari sub county Council, a former forced wife and mother of six CBIC linked to cattle raids, a female councillor, a female neighbour to a CBIC, a religious leader, and a community self-help group leader.

A third source of data which was applied to triangulate information from the FGDs was a set of four Key Informant Interviews (KIIs) conducted with the Community Development Officer of Otuke district, the Chairman Adwari sub county Local Council, the Otuke District Women representative to Parliament, and a social worker from FAPAD based in Otuke. These KIIs were important in exploring and reflecting on norms, perceptions and practices related to CBIC.

The study team was hosted at FAPAD, a Lango-based grassroots organisation active in Otuke whose target groups include victims and survivors of conflicted-related sexual violence and their offspring. FAPAD provided access to its archives on beneficiary information and with guidance from its field based contact persons in Otuke, supported the identification of study participants. Additionally, the authors utilised contact details of CBiC with whom they had engaged in 2019 as part of a creative project for CBIC. All F-PAL study participants received detailed participant information and provided informed consent using the LIREC-approved consent forms translated into the Lango language.

### Generational time among the Lango

Generational time amid the Lango of northern Uganda, among whom the CBIC lived, can be understood as a ‘relational time, enabling sociocultural and political transmission between people of different ages and eras’, in addition to the ‘charting of births, ageing, and deaths’, it also manifests ‘in the construction of symbolic generational orders and metaphors’ [8]. This is because the Lango are typically patrilineal, with its kinship system locating individuals within a relational context that continually shapes their experiences. Among the Lango, relational time has long been marked by acts of rites of kinship, marriage, births, and death interwoven with claims and counterclaims. Using Browne’s [8] understanding of generational time as *relational time* we used our data to explore how time is produced and how it is experienced by CBIC who resettled and lived among their maternal home communities in northern Uganda. As many of the rites of time, in the eyes of the kinship group and by extension also of the CBIC themselves, are compromised by their conception and birth in captivity outside the customary laws and practices, our contribution focusses on the question of how generational time has evolved and is understood for and by CBIC. They and transgenerationally also their offspring are affected by the disruption that comes with conception and birth outside the sanctioned kinship negotiations and the question of the multigenerational impact of this perceived disturbance arises.

Among the Lango, normative patrilineal generational time was marked by the birth of a wife’s offspring, which would, by default, also mark the offspring’s subscription to the husband’s lineage along with the right to associate with its social, cultural, political and economic resources. This article explores how these experiences feed into what the local communities perceive as generational time, and how the prevailing perceptions affect CBIC and community’s responses to being a CBIC or responding to a CBIC. The authors do this by situating CBIC within the broader kinship context in Lango, a society that is patrilineal and exogamous, and who apply marriage as the main lineage-making tools for offspring of daughters (e.g. [4]). The authors found that CBIC in Lango society were frequently perceived as an anomaly in the production of generational time; this impacted negatively on their positionality within local communities, rendering them at a disadvantage.

### The Lango kinship system

As described extensively in the literature, Northern Uganda’s Luo-speaking Lango society, our study location, is typically patrilineal and exogamous, with the kinship system placing individuals into a relational context that is foundational for their social and kinship experiences. Individuals in Lango identify with a clan system, the *atekere* which is traced to a common ancestor. The *atekere* itself is a conglomerate of lineages. The lineage is the lowest unit of association that brings together families across generations [2, 4, 35].

A clan is therefore comprised of groups of lineages or *dogola* (literally, door), headed by a patriarchal figure who, (in principle) often holds in trust the resources associated with his lineage, such as land and cattle. More often than not, he also leads in sanctioning social and cultural aspects associated with his lineage, including marriage, burial ceremonies, mediations, blood feuds, and other forms of compensations and claims such as – and of particular significance in our context - *luk* (illegitimate sexual relations and births outside of marriage). Membership to a lineage therefore provides recognition of an individual’s rights and obligations, which are passed down to subsequent generations as the lineage branches out and differentiates in the complex myriad of (patriarchal) kinship, forming a *generational time* *code* which is acknowledged and traceable at the broader clan level; as a grandson/son grows up to become a father and then a grandfather. In other words, every time a birth takes place, time is produced in that lineage so that the lineage is cast as a representation of time itself. The lineage is therefore an embodiment of time across generations.

For an individual, the embodiment is realised at the time of birth, an event that sets forth a series of other associated events, each of them defining the relationship between the individual (child) and other people and resources associated with the lineage. An individual’s experiences would then generate a series of events subsequent to birth such as enrolment in school, accessing (family/lineage) land and property for livelihood, residing in a home, eating meals, among others. Each of these events can be seen as a point in time ‘where relationships between bodies of materials and people (individuals) come together in intersections’ [30]. In other words, in the case of Lango lineage practices, the birth of an individual to a lineage itself and subsequent life course experiences of the individual are not only a marker of time (generational time code) but also produce time because they are events (cf. [38]).

Therefore, by relying on the event of birth of offspring to code time for the lineage, the Lango do not dwell on chronological time but on one which is defined and nuanced by events as they happen within the normative clan system. When a birth does not take place according to the normative rules (i.e. birth within a clan-sanctioned marriage), there is no coding that takes place (and therefore no production of time), and there will likely be no normative interaction or dialogue between an uncoded offspring and resources linked to a lineage – including land, livestock and people. This is true for CBIC, whose birth in LRA or Karamojong captivity, even if (forced or consensual) marriage had been enacted, would have occurred outside the clan-sanctioned marriage that is part of lineage-making and lineage-shaping. Such a birth would fail to define rights and obligations as the individual (in this case CBIC) reaches different milestones ordinarily defined for bona fide members of a lineage. In other words, the life experienced by a woman’s offspring born within the confined norms of reproduction in Lango (i.e. a sanctioned marriage) produces ‘a rhythm of multiple time flows, with time felt as an element of experience’ [38]. In contrast, the life lived by a women’s offspring born outside these confined norms does not produce these time flows.

### CBIC and generational time

As indicated above, studies on CBIC have explored in depth, what it means to be biologically associated with enemy or rebel soldiers and being conceived in CRSV. For example, children born to girls abducted in northern Uganda (1987–2008) [2, 23], Sierra Leone (1991–2002) [19], or Rwanda (1994) [29] are associated with varying degrees of rejection, discrimination and stigma. CBIC have been found to struggle and frequently fail to integrate into their maternal communities. Their struggles are also often linked to the difficulties their mothers face in reintegrating, themselves often suffering different forms of stigma and discrimination. Accounts from CBIC in our study and those from other key informants show that CBIC experience forms of ‘multiple generation disadvantage’ both at familial ([36], p.1) and local community levels [33]. Akello^3^, one of our interlocutors who was born as a result of CRSV linked to cattle raiders from Karamoja demonstrated the significance of the lineage when a woman’s offspring is born outside of the conducts regulated by norms of reproduction,

My children are treated very poorly in the community. They suffer stigma all the time. They called them ‘*akwar olok*, {grandchild of the nomadic Karamojong cattle raiders}. The neighbours do that all the time. The children are referred to as *akwar olok* (FGD: Female, Acung Apenyi village, Okwang Sub County, Otuke District, Uganda. 5 April 2023).

The study conducted showed that terms such as *akwar alok (grandchild of a cattle raider*^4^*)*, *alok (cattle raider)*, were used by local communities to more broadly refer to offspring of women born of CRSV perpetuated by cattle raiders, and *kony* (in reference to Joseph Kony – the LRA leader) referred to those whose births were linked to the LRA. Interviewees like Akello who were born out of CRSV had these terms extended to their offspring as well, so that the labelling actually went down three generations; Akello’s mother, Akello, and Akello’s offspring.

The literature on CRSV has associated such references to name calling, and have further linked them to rejection, stigma and discrimination [23, 31]. As we will show below, these references; name-calling and associated stigma, in the case of northern Uganda’s CBIC, also contain specific generational metaphors that local communities used to demonstrate the distortions and breakages in their normative generational time.

Metaphors are common in narratives of CBIC and their associates, and they help understand the experiences of CBIC within the local normative generational time. In other words, metaphors such as *akwar alok* typify a brokenness of the child’s lineage, a strangeness of the child, and a distancing of the child from the prevailing lineage of their mother and her marital or familial community. The child is not recognised and is not coded as part of the generational marker in the lineage. It serves as an acknowledgement that the birth of a CBIC was not the normative ‘event’ that produces ‘lineage time’, and that subsequent ‘events’ that ordinarily produced ‘time’ as the CBIC grows and experiences life would also fail to serve as such markers and time codes. Thus, whereas the birth of a child born within the sanctioned generational normative clan system would automatically guarantee the child’s rights to access and interact with lineage resources (bodies and materials associated with the lineage and community), moreover further enhanced by possibilities of future events that would further produce waves of ‘time’ in their generation lineage system, CBIC born outside this normative framework would not have similar rights and obligations. Thus, the luo term, *akwar* or *kwar*, does not only translate to ‘grandchild’, but also has a generational connotation that signifies the importance which local communities attach to the patrilineal identity of a woman’s offspring in reproducing lineage, and therefore generational time.

In the case of Akello’s children, the Lango appropriate their local normative *generational time code* based on their kinship ideas and practices; marked by the birth of offspring a woman begets while married into the lineage/clan of her husband, and successively the offspring the woman’s male children themselves beget upon marriage and so on and so forth. Each of these sets of births within a clan-sanctioned marriage marked the passage of time. A clan leader of the *jo*^5^ Ocukuru Ogora^6^, while referring to his clan explained that his lineage membership was composed of countless generations of offspring of women who entered into marriage with men in his lineage. He referred to both the current and past generations often headed by a senior male head – which made it possible to trace far back and be traced back in the history of his clan. Another elder of the *jo* Palamyek clan^7^ offered that the lineages of his clan rarely had more than three generations living at any given time, and that these were: a patrilineal grandfather (and his brothers and families), father (and his brothers and families), and grandson (daughter) and their siblings. While the length of the generational chain varies, the chains in all cases transmit rights, responsibilities and access to resources to several generations, and – significantly in our context – means that the broken generational time code is transferred from the CBIC to their children, too.

Offspring of unmarried daughters, as in the case of Akello, were considered *atin*^8^*luk*, children born out of illegitimate sexual unions. The illegitimacy could be addressed and resolved through clan negotiations. It could be reversed when the genitor and his family invoked the local *luk* policy to appease the aggrieved family of the child’s mother through payment of a fine of head of cattle (often 1 cow) and other items (goats, hens) mirroring marriage payments. This process would symbolically annul the illegitimacy, reversing the (undesirable) lineage affiliation of the child from that of its mother’s patriclan to that of its genitor (see also [2, 3]).

Importantly, both marriage and *luk* are invoked between families/clans of the ‘suitors’/parents of the child [2]. It is an inter-communal ceremony, which brings together elders from the man and woman’s sides to determine and seal the place of a couple’s offspring in the patrilineage of its genitor, and therefore recognise and code the birth of their offspring as a marker of time in that particular lineage. As a member of that patrilineage, the offspring would be entitled to rights and privileges associated with the lineage. Lineage therefore provides the medium within which members may interact with each other and the associated resources. Significantly, the resolution of the illegitimate birth takes place outside chronological time; and the retrospective sanctioning of the parental union within the cultural norms allows the ‘creation’ of generational time and associated time codes that allow full association with the clan including all the rights and responsibilities.

While possible in principle, in the case of CBIC in northern Uganda, studies have shown that affected families could not easily rely on the *luk* process to support the integration of CBIC either in their maternal or paternal families [2, 4]. Firstly, in many cases CBIC paternity was either not known or not traceable; secondly, and in terms of development and implementation of cultural norms, it is important to note that *luk* was and is conceived as a peacetime policy conducted by the families of both the girl and her suitor/lover before and outside marriage, and is not easily adaptable to wartime where the father of the child does not subscribe to the same norms and rules. Specifically, in most cases, *luk* cannot resolve sanctioning of extramarital relations (and their offspring) in instances of rape and forced marriage by militant groups [4]. Thus, the customary arrangements within the Lango patrilineages effectively establish a framework of permanent exclusion of the CBIC on the grounds of provenance and – due to transgenerational transmission of shortcomings in time codes – for generations to come, too.

In other words, CBIC births were not automatically recognised and coded as a marker of time in any of the lineages associated with their genitors. Rather, and as seen in the example of Akello’s children, the local people used a default generational code built into a mother’s patrilineage as the reference frame to redefine the identity of her offspring – based on peacetime *luk* policy. But in the case of aggression (births whose genitors are linked to violent conflict), Lango lineages distanced their *luk* policy from being invoked as seen with Akello’s offspring, instead perceiving the children as belonging to (the lineage of) Akello’s unknown Karamojong biological father of her offspring and his fathers. Her children were therefore viewed as part of a generation of an unknown Karamojong lineage.

Like Akello, survivors of conflict related sexual violence linked to the Lord’s Resistance Army (LRA) and cattle raiding conflicts in northern Uganda tended to return with their offspring to their pre-conflict familial communities. While some cultivated new conjugal relationships and may or may not have relocated outside lineage confines together with their offspring, others continued to live with maternal patrilineal families whose generational time code was held together by layers of claims and counter claims to economic, cultural and social resources associated with respective lineages [2]. 

This outlook did not only portray the children as ‘lost’ in terms of generational time, but as situated within a generational time code that’s broken or weakened, and with it opportunities to claims and counter claims which would further mark and produce time. In other words, the concept of generational time in Lango society is shaped by the coding of births of new-borns, which, by construct, did not provide for CBIC. Driberg [14], writing in 1923, observed a number of *kwer*, ceremonial rites associated with newborns, including naming which can be seen as a form of coding; naming of the child, he wrote, followed some kind of pattern, ‘stereotyped by custom’, with a first-born called after his father’s father, and subsequent children named after brothers of their fathers, and then paternal grand uncles, in that order (pp. 141–148). CBIC, in most cases, could not benefit from such rites, some of which – like the naming – still exist, as they entered their maternal Lango lineages without the paternal reference points arising out of a sanctioned marriage.

This outlook also marks them out as different to the rest of the community whose lineages are traceable and known – and often come with established resources, such as land, for claims and counterclaims. It casts CBIC as outsiders, as aliens, whose parentage dislocated them from potential claims to ‘privileges and immunities’ of members of the lineages their mothers associated with – often referred to as discrimination in the literature.

### Distortion of sense of generational time

Not knowing a biological father or at least his lineage meant CBIC could neither contribute to nor draw on the generational time code of that unknown lineage to base any claims and counterclaims. Individuals in Lango society relied on their lineage identity to determine where a child could rightfully live, what name that child was given, on whose lands that child could have usufruct and inheritance rights, who would have responsibility for providing for the child and protect that child growing up, the source of the child’s brideprice (in the case of male offspring), and what opportunities the child could have in their life course [4]. For a female child, the lineage affiliation would determine who had say and stake over transfer of her sexuality rights and marriage goods in adulthood [4]. In other words, lineage membership spelt out new rights and privileges, including the right to physically affiliate to and live with a family, in a household, and to inherit and benefit from resources associated with members of that lineage. The fact that CBIC could not fulfil the local lineage affiliation policies through birth ultimately created a form of ‘lineage’ vacuum, a generational ‘time gap’ for them.

This ‘time gap’ cut them off the paternal generational timeline and distorted the sense of generational time for CBIC, exposing them to a lack of avenues to contribute to the production of waves of time and their meanings. The sense of distortion was expressed and experienced variously, including through denial of usufruct rights to land by maternal family and neighbours. For example, Acen stated,

…even when it comes to resources, to things, no one will allow you to access or touch it. Even when you go asking for a mortar {for pounding cereals} like this, they will not accept. This common practice of passing things around within the neighbourhood, it is not happening with me. Worse, when you send out a child, to go fetch something, no one will accept {to associate or lend}. Even a portion of land for tilling, they will not accept, if you move to ask to use it temporarily to plant even just potatoes, no one will accept. They will say, Aaah! You will bring ‘olok’ {cattle raiders from Karamoja} to raid us, to grab our land, when we get involved, when we get used to you. You will bring ‘olok’ here because you are one of them. (FGD: Female, Te-yao village, Adwari sub county, Otuke district, Uganda. 19 April 2023).

Acen’s example illustrates how the sense of distortion of generational time for CBIC is relational and intersects with perceived rights within the lineage and neighbourhood. In Acen’s local community of Otuke, the pathway to rights within her mother’s patrilineage was charted alongside the generational code borne by an individual. If an individual was perceived as not belonging to a lineage, then the individual had no direct rights and privileges associated with that lineage (unless the ceremony of *luk* was sanctioned), and any ‘events’ associated with them could not count in producing time in that lineage and community. In other words, by not being a marker and therefore producer of ‘lineage’ time, the CBIC would not have any generational contract associated with the lineage and therefore would not lay claims and counter claims on that lineage – which events would otherwise lead to the further production of time through their relationships with ‘materials and bodies’ associated with the lineage/community. Importantly, this addresses the question of whether CBIC have rights where they do not have any generational contract with the lineage, and whether any denial of such ‘rights’ would amount to discrimination and stigma for the CBIC.

Our interlocutors cited many examples where they were unable to access support ordinarily extended to members of their mothers’ relations on account of their not being coded in the lineage. A female CBIC, Stella, for example stated that,

For me, ever since I was told that my father is a Karamojong (cattle raider), I wasn’t even sent to school, I am not treated the way other children at home are treated. They say my father is different so this has made my life hard…They say that I’m a bastard without a father who was born in the bush. It made me cry someday that if I had my father, I would have studied (FGD: Female, Barlwala Village, Okwang Sub County, Otuke district, Uganda. 5 April 2023.).

Isacc, a male CBIC cited a similar experience;

People do not treat me well at home compared to the other children. For example, my brother with whom I sat for Primary Leaving Examination got aggregate 19 {and} was taken to study in Adwari senior secondary school, but I am not studying now and yet I got aggregate 18. Although he is older than me I asked him why he was going to school and I am not, my uncle just told me to remain home (FGD: Male, Miciri village, Okwang Sub County, Otuke District, Uganda. 5 April 2023).

Our interlocutors also cited the impact of the brokenness of the sense of generational time on CBIC’s residential right as compared to those coded. In many cases, CBIC who did not have own independent abodes faced rejection and abuse. For example, Akullo, a female CBIC, stated that,

…the person we called father {her stepfather} keeps saying that, ‘now this dog has again arrived’, that he does not wish to see me, that I should be taken away… My mother would refuse, saying she was willing to die with her child next to her, and that I should be allowed to stay. So, I have continued to live there with a lot of suffering. One {farming} season, when he allowed me to plant my sunflower on a patch of field, he waited for the harvest and grabbed it all for himself {FGD: Female, Odokomit, Adwari sub County, Otuke District, Uganda. 19 April 2023}.

CBIC who had stepsibling not necessarily fathered by cattle raiders and the LRA, but whose biological fathers were known and traceable stated that their stepsibling had better relationships with their mothers’ maternal and marital families. The explanations often linked this favourable relationship to the fact that the other stepsiblings could easily be associated with the old *luk* policy that by default defined ways of coding offspring born outside of marriage to a lineage. CBIC were not perceived as children of *luk* [4] and could therefore not benefit from its generational coding policies. For example, the female CBIC Akullo, stated that,

As for my half-brother, he is treated well compared to me. This is because people say for him, his father is known, and he can easily be taken to his father {by uttering the policy of *luk*}. He {stepfather} told me that he has nothing at all {for me}, and so people would keep telling me to look for my father, and yet I don’t really know (FGD: Female, Acungapenyi Village, Adwari sub county, Otuke district, Uganda. 19 April 2023).

Some CBIC stated that they experienced physical attempts to eject them out of the family/community they lived in on account of not being a part of the lineage. For example, a female CBIC stated;

…they would side with what my {maternal} uncle would say. That I am now a grown up, I should go look for my father. That if I do not look for my father, I should consider eloping {with a man}. The anger I have because of this keeps me away from {that} home {cries} (FGD: Female, Acungapenyi village, Adwari Sub County, Otuke district, Uganda. 19 April 2023).

### Children conceived in peacetime but born in captivity

The experiences of CBIC were not any different for children who had already been conceived before their mothers were abducted and were therefore born either in captivity or upon the return of their mothers after a short period in captivity. These children, summarily referred to also as CBIC had similar experiences of generational time distortion and were neither acknowledged as part of the markers on the generational time code nor as bona fide beneficiaries of the resources of any of the lineages they associated with. For example, Odongo, a male participant stated,

When we came back from the bush I was still young, we stayed shortly and ‘my father’ who had married my mother died. But how I used to live, people would say it’s a Karamojong cattle raider who fathered me, and yet she went there with me before I was born, mother was already pregnant with me, she went with me there {into captivity} (FGD: Male, Kamdini village, Adwari sub county, Otuke district, Uganda. 19 April 2023).

Overall, distortions in generational time for CBIC had far-reaching consequences on the relational aspects that ordinarily defined the social ecologies within which CBIC lived. Although the Lango is a society in flux, their daily lives continue to be defined by communal undertakings where families and neighbourhood closely interact with each other to produce time.

### Multigenerational distortion

The sense of distortion in generational time appears to be multigenerational, with children of CBIC having similar experiences as, for example, cited by Adongo, a female CBIC,

I then went to hospital to have my child. When I came back home with my baby, I was shunned by people in the community. People would say, ‘who on earth feels like visiting the newborn ‘akwar olok’ {grandchild of cattle raiders). But I lived. And as my child grew older, he could not cross any path or road in the neighbourhood without being abused. Children in the neighbourhood would chase him back home. He would run back home crying as those in the neighbourhood shout out ‘akwar olok, akwar olok’ (FGD: Female, Akailor Village, Adwari sub county, Otuke district, Uganda. 19 April 2023).

Interlocutors cited discrimination, stigma and deprivation as common experiences, which both CBIC and their offspring experienced. As happened with CBIC, their offspring were most often not recognised as part of a marker on the generational code of a lineage – even where their CBIC mothers and fathers had them in a marriage. A female CBIC, for example, drew on her case, stating;

These days, even when I am just seated {peacefully} other people would be pointing at the back of my head {stigmatising me}, accusing me of being ‘the child of ‘alok’, that; ‘this child belongs to ‘alok’. Even all of the children I have given birth to, people refer to them as children of olok’… (FGD: Female, Akailor Village, Adwari sub county, Otuke district, Uganda. 19 April 2023}.

Such a continuum from parent to offspring suggests that once an individual is not seen as a marker of a lineage’s generational time code, the individual as well as their future offspring drops off the lineage and will have difficulties intersecting with lineage materials and bodies that enable members generate lineage time. In other words, both CBIC and their offspring cannot easily access and freely relate with the cultural, social, economic and political resources that are a preserve of the lineage and community. For example, participant Adongo further stated,

…my children now have a difficult life. Even when they are in school, they live in fear…my child actually comes to me and says, ‘mother, were you really born in the bush? Why would people say to me that my mother came from the bush?’ That ‘mother, were you really born in the bush…? (FGD: Female, Akailor Village, Adwari sub county, Otuke district, Uganda. 19 April 2023}.

A similar account was given by male CBIC who were married and had children of their own. For example Okello, stated;

When I am home, people call me *Ojong* {Karamojong}. Even my children are referred to as *Ojong*, and I don’t understand why. I feel bad, because people do not like the *Ojong* {because they are cattle raiders}, I feel such a name should not be attached to me or my children. But also I see that some people call me that to ridicule and abuse me (FGD: Male, Otumpiri village, Okwang Sub county, Otuke District, Uganda. 5 April 2023).

The use of the term *ojong* to refer to CBIC and their offspring is another of the generational metaphors discussed above. The Karamojong are a different language group and lineage, and the event of the birth of the CBIC, and later his children, could not be seen as markers of the generational time code of the CBIC mother’s lineage. The CBIC and his offspring could not therefore easily claim rights and freedoms which shape events that continuously produce time in that lineage.

### CBIC ‘fighting back’

It is important to note that CBIC were often not merely passive observers or victims of the normative functions of time-producing activities of lineages their parents associated with. Instead, experiences of some CBIC further showed the different ways that both the CBIC and those they interacted with were often ‘fighting back’ to reorganise and claim rights and privileges as markers and producers of generational time. These ranged from subtle forms of silence and emotional responses to physical actions including seeking help from local leaders.

For example, Okello, a male CBIC stated that when his stepfather refused to pay his fees and he dropped out of school, he approached a local leader,

I took my complaint to the youth leader, and told him I wanted to go back to school. He then mobilised other elders and they came and talked to him {stepfather}. But he refused. He said he had many problems to solve, and moreover he had already raised me, and I had already become an adult. I should therefore find my ‘level’ (FGD: Male, Agwila Village, Adwari sub county, Otuke district, Uganda. 19 April 2023).

While Okello’s objective of going back to school through the local leader’s intervention was not met, another CBIC successfully lodged his complaint by invoking the intervention of local leaders. For example, Ogwal stated that,

You know we as youths are stubborn, we went for a dance and one day I came home with a woman and stayed with her inside the house and then my {step}father’s brother told me that I should go and look for my father to come and pay that woman’s bride price. I went and reported to a {local} leader who gave me a letter to go with to police and he was arrested (FGD: Male, Acungapenyi Village, Adwari sub county, Otuke district, Uganda. 19 April 2023).

Some CBIC stated that they were often emotionally affected and became silent in anger. For example, a male CBIC, Otim stated;

Sometimes I feel sad, very sad but sometimes I also live care freely. After thinking about it and my heart calming down and then the anger goes, I start being carefree. I don’t show him that I’m angry, but I’d be angry, but I just don’t show that I’m angry, I stay silently (FGD: Akailor Village, Adwari sub county, Otuke district, Uganda. 19 April 2023).

Another CBIC stated that he was never quiet and would confront those stigmatising him. He recalled,

I did not, I would tell them I am not *ojong*. I have another problem, which is stigma, for example, people call me names and I don’t like it, they say I was born by the Karamojong and that I will die with AIDS, because I am on medication (‘he normally gets mental health treatment from Lira mental health unit at Aki Bua’) (FGD: Male, Otumpiri village Okwang sub county, Otuke district, Uganda. 5 April 2023).

Another, a female CBIC, explained that she relied on her Christian faith to confront those who discriminate her. She stated,

There came a time when things became really tough, and my heart had to just let go. Firstly, I said to God, there is now nothing I can do. I have already been born, and can’t climb back into my mother’s womb. Please give me another option. And as I mentioned earlier, I went to a religious leader, I cried to him, because I had reached a point where I had given up… (FGD: Female, Acung Apenyi village, Adwari Sub county, Otuke District, Uganda. 19 April 2023).

Other CBIC simply acknowledged the brokenness in the continuity of generational time associated with their mothers (and most often unknown fathers), and quietly relocated to other places where their background would not be known.

The man my mother is with abuses my mother for everything all the time. He is very tough. If my mother picks money to pay for my studies, he beats my mother badly and when my grandmother died, I cried so much and I wanted to die as well because I wasn’t getting any peace in the world but I persevered. My uncle also doesn’t really like me but I persevered. Then I left to come and be with my mother but the man doesn’t want to see me and only takes care of his children. He doesn’t want to see me with anything and even if he buys anything he doesn’t give me to eat. I only have peace when my mother comes back because by then he is already drunk. Anger made me escape and go to the home of my mother’s sister and I found that she has the same kind of heartache and I just started cohabiting with a man (FGD: Female, 24 years old, Akano village, Okwang sub county, Otuke district, Uganda. 5 April 2023).

## Conclusion

Whereas the current article contributes more broadly to the understanding of temporalities among African societies, it more specifically explores how a post conflict African patriarchal society finds its normative frame for producing time through marriage, childbirth and associated relational events of living, challenged by births associated with CRSV. Our focus is on generational time in terms of a CBIC temporal situatedness within the Lango people’s generational kinship system. Our findings suggest that CBIC’s experiences of what practitioners and policy makers typically refer to as ‘stigma and discrimination’ are principally rooted in (kinship) community perspectives of a failed temporal locatedness in the intergenerational social fabric of the local community.

The birth of CBIC was an anomaly to the local lineage affiliation policies in patriarchal Lango society, where each lineage was hardwired with a default generational code, which was applied as the reference frame for defining and redefining an offspring. When they did not meet the requirements of the jural rules as indeed was perceived of the CBIC, then they became an anomaly. Their births could not be used to code and mark lineage time, and yet this coding was buttressed with the rights to claims and counter claims – resources such as naming, usufruct and inheritance rights to land, right to live in a family, right to provisions for growth and protection into adulthood, source of bride price, among others. In the case of the 22 CBIC study participants in their FGDs, CBIC were not only portrayed as ‘lost in terms of generational time, but as situated within generational time codes that were broken, or weakened, and with it the CBIC’s opportunities to claims and counterclaims associated with lineages, and everyday actions which would further produce and mark time.

These, among other things, gave rise to stigmatising labels, which were actually metaphors that typified a brokenness of the child’s lineage, a strangeness of the CBIC, and a distancing of the CBIC from the prevailing lineage and resources associated with their mothers/fathers. These metaphors referred to a ‘time gap’ because of failed coding in lineage time. Practitioners and policy makers can therefore redefine and align their understanding of ‘stigma and discrimination’ to local cultural and temporal considerations. Additionally, this article makes a first contribution to aspects of CBIC’s experiences in terms of temporalities associated with CRSV. Future studies can consider comparative explorations across cultures, conflicts and societies to further understand variations in temporalities and how they might impact on local CBIC. Scholars might also be interested in examining how gender intersects with CBIC situatedness to shape distinct temporal experiences for CBIC and their communities. Importantly, this article argues for more in-depth understanding of CBIC experiences of so-called ‘stigma and discrimination’ by re-situating CBIC (and their claims and counter claims) within the temporal threads of their local communities.

## Data Availability

The datasets generated and analysed during the current study are not publicly available, as the study is still ongoing, but they are available from the corresponding author on reasonable request.

## Abbreviations

CBIC: Children born in captivity
CBoCRSV: Children born of conflict related sexual violence
CRSV: Conflict related sexual violence
F-PAL: Film in Participatory Action Learning
FGD: Focus group discussions
LRA: Lord’s Resistance Army

## Footnotes

1 Interview with Angelina Acheng, founder of Concerned Parents Association, Lira, Uganda. 04 November 2025.

2
https://rights4time.com/. AHRC Global Network Plus Rights for Time, grant reference number AHRC 1,000,834.

3 All names are pseudonyms to protect participants’ confidentiality.

4 Alok is a derogatory reference to cattle raiders from Karamoja region.

5 The prefix *jo* typically means *‘people’*, in this case; the people of the Palamyek clan.

6 KII: Muzee Donga Peter, Aputi village, Abeli Parish, Akalo Sub County, Kole district, Uganda. 28 January 2024.

7 KII: Aremo Charles, Aol Kedi village, Acokara Parish, Otwal Sub County, Oyam District, Uganda. 27 January 2024.

8
*Atin* means ‘child’, in this case; child of *luk*.

## References

[1] Annan J et al. Women and girls at war: ‘Wives’, mothers and fighters in the lord’s resistance army. Househ Confl Netw Paper, 2009;63.

[2] Apio EO. Resilience among children born of war in Northern Uganda. Front Polit Sci. 2022;4:874548. 10.3389/fpos.2022.874548.

[3] Apio EO. Children born of war in Northern Uganda: Kinship, marriage, and the politics of post-conflict reintegration in Lango society (Doctoral dissertation, University of Birmingham). 2016. https://etheses.bham.ac.uk//id/eprint/6926/1/Apio16PhD.pdf

[4] Apio EO, Gender Kinship, and affiliation of children born of war in patriarchal Northern Uganda. In: Theidon K, Mazurana D, Anumol D, editors. Challenging conceptions: children born of wartime rape and sexual exploitation. New York: Oxford Academic; 2023. 10.1093/oso/9780197648315.003.0005.

[5] Arasio RL, Stites E. The return of conflict in Karamoja, uganda: community Perspectives. Karamoja resilience support unit (KRSU), Feinstein international center. Kampala, Uganda: Friedman School of Nutrition Science and Policy at Tufts University; 2022.

[6] Baines E. Forced marriage as a political project: sexual rules and relations in the lord’s resistance army. J Peace Res. 2014;51(3):405–17.

[7] Baines E, Oliveira C. Securing the future: transformative justice and children ‘born of war’. Social Legal Stud. 2021;30(3):341–61. 10.1177/0964663920946430.

[8] Browne V. Generational time. Feminism, Time, and nonlinear History. Breaking feminist waves. New York: Palgrave Macmillan; 2014. 10.1057/9781137413161_6.

[9] Carpenter C. Forgetting children born of war: setting the human rights agenda in Bosnia and beyond. Columbia University; 2010.

[10] Carpenter RC, editor. Born of war: protecting children of sexual violence survivors in conflict zones. Kumarian; ed., 2007.

[11] CEDAW-CRC. CEDAW-CRC joint statement: Ensuring prevention, protection and assistance for children born of conflict-related rape and their mothers. 2021. https://www.ohchr.org/en/documents/statements/cedaw-crc-joint-statement-ensuring-prevention-protection-and-assistance

[12] Denov M. The meaning of land and place for children born of war in Northern Uganda. Children’s Geographies. 2023;21(4):693–707. 10.1080/14733285.2022.2113857.

[13] Denov M. Children born of wartime rape: the intergenerational realities of sexual violence and abuse. Ethics Med Public Health. 2015;1(1):61–8. 10.1016/j.jemep.2015.02.001.

[14] De Nutte L, De Haene L, Derluyn I. They now know that they are children of war: forcibly abducted mothers and fathers balancing disclosure and Silencing to their children born of war in Northern Uganda. Front Polit Sci. 2022;4:850969. 10.3389/fpos.2022.850969.

[15] Driberg JH. The lango: A Nilotic tribe of Uganda. London: T. Fisher Unwin Ltd. [Pdf] Retrieved from the Library of Congress; 1923. https://www.loc.gov/item/2021666779/.

[16] Foreign C, Development Office. Ensuring the rights and wellbeing of children born of sexual violence in conflict: Call to action. 2021. https://www.gov.uk/government/publications/ensuring-the-rights-and-wellbeing-of-children-born-of-sexual-violence-in-conflict-call-to-action/call-to-action-to-ensure-the-rights-and-wellbeing-of-children-born-of-sexual-violence-in-conflict

[17] Foreign C, Development Office. Platform for action promoting the rights and wellbeing of children born of conflict-related sexual violence. 2022. https://www.gov.uk/government/publications/platform-for-action-promoting-rights-and-wellbeing-of-children-born-of-conflict-related-sexual-violence/platform-for-action-promoting-the-rights-and-wellbeing-of-children-born-of-conflict-related-sexual-violence

[18] Foreign C, Development Office. UK calls for global action to end the stigma faced by children born of sexual violence in conflict. 2021. https://www.gov.uk/government/news/uk-calls-for-global-action-to-end-the-stigma-faced-by-children-born-of-sexual-violence-in-conflict

[19] Foussiakda CA, et al. Children born of conflict-related sexual violence: A review of interdisciplinary responses to their needs and experiences. J Hum Trafficking Enslavement Conflict-Related Sex Violence. 2023;4(1):59–82.

[20] Grieg K. The war children of the world. War and Children Identity Project Bergen. 2001. https://www.academia.edu/2189623/The_war_children_of_the_world

[21] Gustavsson M, Oruut J, Rubenson B. Girl soldiers with lord’s resistance army in Uganda fighting for survival: experiences of young women abducted by the LRA. Children’s Geographies. 2017;15(6):690–702. 10.1080/14733285.2017.1300233.

[22] Kiconco A. Gender, conflict and reintegration in uganda: abducted girls, returning women. Taylor & Francis; 2021.

[23] Kiconco A. Children born of rebel captivity: politics and practices of integration in Uganda. Front Political Sci. 2022. 10.3389/fpos.2022.823995. 4.

[24] Kiconco A, Nthakomwa M. Marriage for the ‘New woman’ from the lord’s resistance army: experiences of female ex-abductees in acholi region of Uganda. Women’s Stud Int Forum. 2018;68:65–74. 10.1016/j.wsif.2018.02.008.

[25] Lee S. Children born of war in the twentieth century. Manchester University; 2017.

[26] Sabine L. Unintended Consequences or Desired Outcome? In: Challenging Conceptions. Edited by: Kimberly Theidon, Dyan Mazurana, and Dipali Anumol, Oxford University Press. 2023;56–86. 10.1093/oso/9780197648315.003.0004

[27] Lee S, Glaesmer H, Bartels SA. Children born of war: Challenges and opportunities at the intersection of war tension and post-war justice and reconstruction. *Frontiers in Political Science*, 2023;5. 10.3389/fpos.2023.1122280

[28] Madhani PD, Baines E. Fatherhood in the time of war and peace: the experiences of demobilised male soldiers in Northern Uganda. Women’s Stud Int Forum. 2020;83(November–December 2020):102415. 10.1016/j.wsif.2020.102415.

[29] Nyirandamutsa F, et al. Perceptions of the intervention utility and effectiveness in supporting and reintegrating youths born of genocidal rape in Rwanda. Adolesc Health Med Ther. 2023;14:141–51. 37720485 10.2147/AHMT.S412300 PMC10505046

[30] Richardson J, Walker S. Processing process: the event of making Art. Stud Art Educ. 2011;53(1):6–9.

[31] Seto D. No place for a war baby: the global politics of children born of wartime sexual violence. Routledge; 2013.

[32] Stites E. Conflict in karamoja: A synthesis of historical and current perspectives, 1920–2022. Karamoja resilience support unit (KRSU), Feinstein international center. Kampala: Friedman School of Nutrition Science and Policy Tufts University; 2022.

[33] Tanton R, Gong C, Harding A. Multiple generation disadvantage: how communities affect the outcomes of different generations. NATSEM, University of Canberra; 2011.

[34] Theidon K, Mazurana D, Anumol D, editors. Challenging conceptions: children born of wartime rape and sexual exploitation. Oxford University Press; eds, 2023.

[35] Tosh J. Clan leaders and colonial chiefs in lango: the political history of an East African stateless Society, c. Oxford: Clarendon; 1978. pp. 1800–939.

[36] Vinson T. Intergenerational disadvantage. Canberra: Australian Department of Education, Employment and Workplace Relations. 2009.

[37] Wagner K, et al. The immediate and Long-Term Risks, harms and challenges faced by children born of Conflict-Related sexual violence (CBoCRSV) in Low- and Middle-Income countries (LMICs): A rapid evidence assessment. University of Birmingham; 2024.

[38] Wapenaar K. ENTANGLEMENTS OF TIME. Int J Child Youth Family Stud. 2015;5(42):826–46. 10.18357/ijcyfs.wapenaark.5422014.

